# Short-term outcome of surgical arthrodiastasis of the ankle with Ilizarov frame in a cohort of children and young people with juvenile idiopathic arthritis

**DOI:** 10.1093/rap/rkz031

**Published:** 2019-08-14

**Authors:** Gavin Cleary, Clare Pain, Liza McCann, Kamran Mahmood, Steven Brookes-Fazakerley, Simon Robinson, Roger Walton, Alan Highcock, Caren Landes, Nik Barnes, Ian Roberts, Leroy James

**Affiliations:** 1 Alder Hey Children’s Hospital NHS Foundation Trust, Liverpool; 2 Arrowe Park Hospital, Upton, Wirral, UK

**Keywords:** ankle, arthrodiastasis, juvenile idiopathic arthritis, pain, functional outcome

## Abstract

**Objectives:**

Despite medical advances, life-changing articular damage may still occur in patients with JIA. We report a cohort with destructive arthropathy of the ankle treated by surgical arthrodiastasis.

**Methods:**

Eight patients (nine ankles) received arthrodiastasis by means of an Ilizarov frame between 2009 and 2013. Patient- and clinician-reported outcome measures were collated prospectively, with retrospective analysis of demographics, disease and pre-surgical treatment.

**Results:**

Pre-surgery, all patients received IA CS (mean 0.8 injections/year) and MTX (mean diagnosis to treatment 3.8 years; two of eight started within 3 months). Seven of eight patients received biologic drugs. Pain scores improved by 56 and 29% (*P *<* *0.005) at 6 and 12 months post-frame removal. American Academy Orthopaedic Foot and Ankle Society ankle–hindfoot scale, Oxford Ankle Foot Questionnaire-Child and Oxford Ankle Foot Questionnaire-Parent scores improved by 171, 62 and 80%, respectively (*P *<* *0.005) at 12 months post-frame removal. Patients remained satisfied with surgical treatment for a mean of 13.3 months. There was transient pin site infection in three patients, and all patients had radiological improvement in joint space.

**Conclusion:**

Arthrodiastasis with an Ilizarov frame is a safe, well-tolerated technique that should be considered as a short-term joint-preserving procedure to improve pain and function when damage has occurred. Delays to systemic medical treatment in this cohort would be considered out-with standard modern practice but, although less prevalent, destructive ankle arthropathy continues to occur in JIA, and we believe this study to be relevant. The ankle is particularly susceptible to damage and, even if localized, should be treated early and aggressively with DMARDs and rapid progression to biologic therapies.

**Levelof evidence:**

Level IV.

**Key messages**

- Arthrodiastasis is safe and effective in the short term for destructive arthropathy in JIA.
- Consider early treatment with a DMARD and escalation to a biologic for ankle and sub-talar disease.

## Introduction

JIA is historically associated with destructive arthropathy [1]. Medical treatment technologies for JIA have evolved considerably in recent decades. In the late 1980s, intra-articular corticosteroid (IA CS) injection and DMARDs, in particular MTX, became established practice. From the late 1990s, the use of biologic drugs became standard, supported by robust evidence from global clinical trials [2]. Despite this, high disease activity 1 year post-diagnosis was reported in approximately one-third of a UK prospective inception cohort, compounded by a persistent delay in access to paediatric rheumatology care [3]. There is widespread evidence of JIA patients experiencing persistent disease activity into adulthood; therefore, the risk of articular damage may be lifelong [4]. In one study, 11% of adults reported severe disability [5]. Some patients with JIA will therefore require orthopaedic treatment owing to arthropathy during the lifetime of their disease.

### Arthrodiastasis in destructive joint disease

Active inflammation, trauma, sepsis and degenerative joint disease may destroy cartilage. Surgical options have previously been limited to debridement, arthrodesis or total ankle arthroplasty. Debridement, although joint preserving, is a temporary measure with poor long-term outcome and OA of the ankle [6]. Arthrodesis and arthroplasty are joint-sacrificing procedures that, in the adult population post-trauma and in RA, have been shown to provide good to excellent pain and functional outcomes in the intermediate to long term [7, 8]. Both are irreversible and associated with serious and long-term complications [9].

In children and young people, preservation of the native ankle joint as a functional, pain-free joint for as long as possible is desirable. Arthrodiastasis is a joint-preserving option. The physiology of arthrodiastasis combines ankle joint distraction with weight-bearing to create intermittent positive and negative IA pressures, which stimulate proteoglycan synthesis [10], decrease mononuclear inflammatory cell inhibition of proteoglycan synthesis, decrease catabolic cytokines, such as IL-1 and TNF-α, and increase delivery of nutrients to chondrocytes [11]. Other theorized benefits of distraction of the ankle joint include its positive effects on capsule nerve endings, decreased subchondral sclerosis, and therefore, better shock-absorption capabilities of the bone, causing decreased joint reactive forces [12].

The efficacy of ankle arthrodiastasis in end-stage JIA arthropathy has never been described. We present a case series of children and young people with JIA who underwent arthrodiastasis and report patient- and clinician-derived outcomes, including pain, measures of function and survivorship of the procedure in the short to medium term.

## Methods

We collected patient- and clinician-reported outcomes measures according to our routine practice, in a cohort of patients who underwent ankle joint arthrodiastasis between 2009 and 2013. The indication for arthrodiastasis was painful destructive ankle arthropathy secondary to JIA. All patients were treated surgically by one of two consultant orthopaedic surgeons at Alder Hey Children’s Hospital, Liverpool, UK. Medical care was delivered by a specialist paediatric rheumatology team. Exclusion criteria were IA infection, hindfoot instability and psychological factors that would not permit a 3-month period of external fixator joint distraction. Retrospective review of the case records collected demographic, disease-related and medical treatment data up to the time of the surgical procedure.

**Fig. 1:**
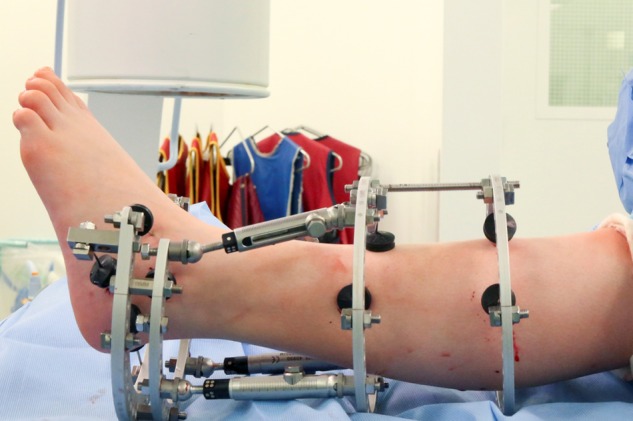
Clinical photograph of standard ring external fixator applied with TrueLok Rapid Struts, providing fixed ankle joint distraction

The operative procedure was performed under general anaesthetic, with the patient supine. A thigh tourniquet was applied, and the leg was prepared with chlorhexidine before draping. A ring external fixator was applied using the TrueLok Ring Fixation System (Orthofix, Verona, Italy). The tibial frame was applied with two rings perpendicular to the longitudinal axis of the tibia. The rings were then connected to the tibia using two 1.8 mm olive wires per ring, tensioned at 50 kg. The foot frame was applied consisting of two 5/8 rings. The rings were held in line with the longitudinal aspect of the foot, aligning the superior-most ring parallel with the talus and ensuring that the inferior ring had enough clearance from the ground to allow weight-bearing. The inferior 5/8 ring was attached to the os calcis with two 1.8 mm olive wires tensioned to 40 kg. A smooth 1.8 mm wire was passed through the talus, connected to the superior 5/8 ring and tensioned to 40 kg. The foot and tibial constructs were connected using four TrueLok Rapid Struts (Fig. 1) before acutely distracting the tibiotalar joint by ∼5 mm as assessed using intraoperative fluoroscopy.

Post-operative care involved daily pin site dressings with chlorhexidine-soaked gauzes until sites were dry, followed by weekly dressing changes. Full weight-bearing post-operatively was allowed. The external fixator was removed after 3 months.

Multiple patient-reported outcomes were assessed. Pain was scored by the patient completing a 10 cm visual analog scale of pain. Subjective patient functional scoring was completed via the Oxford Ankle Foot Questionnaire-Child (OxAFQ-C) and Oxford Ankle Foot Questionnaire-Parent (OxAFQ-P). This is a valid and reliable scoring system marked out of 60 by both the child and the parent [13]. The American Academy Orthopaedic Foot and Ankle Society (AOFAS) ankle–hindfoot scale (maximum score of 100) provided a combined subjective and objective score [14]. Pain scores were measured pre-operatively, at the time of frame removal and thereafter at 6 and 12 months post-frame removal. The AOFAS ankle–hindfoot and OxAFQ scores were measured pre-operatively and at 12 months post-frame removal. We also recorded post-operative complications and survivorship of the native ankle post-frame removal, with the end-point being repeat arthrodiastasis, arthrodesis or total ankle arthroplasty.

Parametric statistics (Student’s two-sided paired *t*-test) were used to compare differences between pre- and post-operative scores. *P*-values at the 5% level or less were considered statistically significant. Calculations were measured using SPSS version 17 for Windows (SPSS Inc., Chicago, IL, USA).

## Results

### Demographics and clinical features

Nine ankles in eight patients were studied. There were seven female patients and one male, with a mean age of 14.3 years (range, 8.7–17.2 years) at the time of surgery. Surgery was performed between 2009 and 2013. The mean time from diagnosis of JIA to surgical arthrodiastasis was 9.4 years (range 5–16 years). There was a trend over time for surgery at an earlier age.

The range of movement of the ankle and subtalar joints was not recorded specifically, but all joints had a grossly restricted range, and most were associated with marked crepitus and pain.

Demographics, JIA sub-type data and a summary of medical and surgical interventions are presented in Table 1.

**Table 1:** Demographic and medical treatment data pre-surgery

Patient characteristics	
Female	7
Male	1
JIA subtype	
Persistent oligoarthritis	2
Extended oligoarthritis	3
Polyarthritis RF Neg	2
Polyarthritis RF Pos	0
Psoriatic arthritis	1
ANA^+^, *n* (%)	5/8 (62.5%) 1 unknown
Age at JIA onset, mean (range), years	2.4 (1.5–4.75)
Age at diagnosis, mean (range), years	3.4 (1.5–6.25)
Clinical data before surgical arthrodiastasis	
Anatomical site	
Left ankle	4
Right ankle	5^a^
Soft tissue calcification, *n* (%)	8 (100%)
Age at arthrodiastasis, mean (range), years	14.3 (8.7 − 17.2)
Time from JIA diagnosis to arthrodiastasis, mean (range), years	10.9 (6.75 − 17.16)
Number of IAI to arthrodiastasis joint pre-surgery, mean (range)	5.6 (2 − 10)
Number of IAI to arthrodiastasis joint per year, mean (range)	0.61 (0.12 − 1.48)
Time from JIA diagnosis to MTX, mean (range), years	3.84 (0.18 − 7.08)
Time from JIA diagnosis to first biologic^b^, mean (range), years	6.44 (4.42 − 11.92)
Number of biologics before arthrodiastasis, mean (range)	1.66 (0 − 3)

a Two patients had bilateral arthrodiastasis.

b One patient did not receive a biologic pre-arthrodiastasis. IAI: Intra-articular injection.

### Radiological findings

**Fig. 2:**
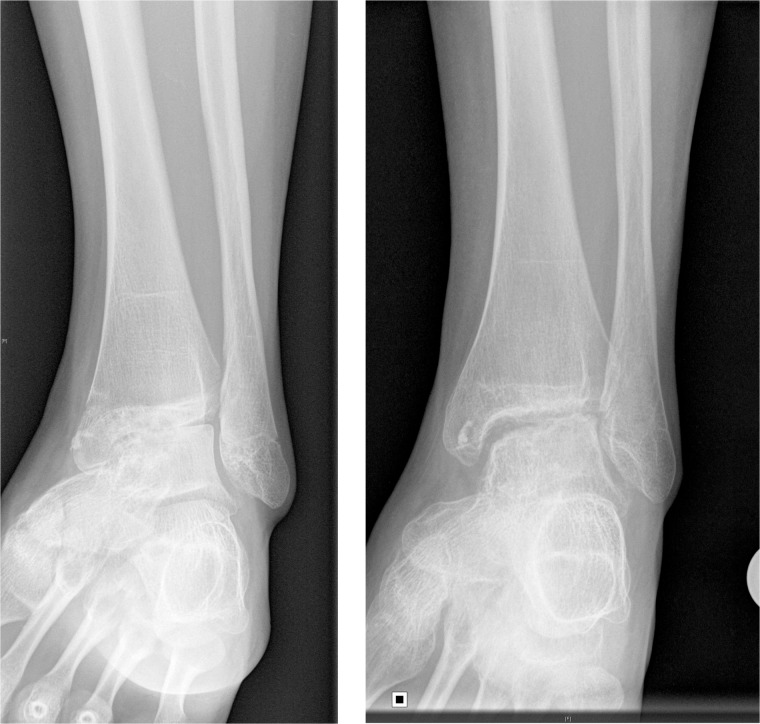
Plain radiographs illustrating increased joint space (pre-operative on left and post-operative on right).

A typical sequence of destructive changes was seen, with loss of joint space and progressive anterior subluxation of the talus. Extra-articular soft tissue calcification was seen in all patients secondary to IA CS injections with triamcinolone hexacetonide (TH). This occurred despite our practice to guide needle placement for IA CS injection by radiological screening. An increased joint space post-arthrodiastasis was seen in all patients (Fig. 2).

### Medical treatment pre-arthrodiastasis

The mean number of IA CS injections to the ankle requiring surgical arthrodiastasis was 5.6 (range 2–10). The mean number of joint injections per year was 0.61 per year (range 0.12–1.48). Five out of eight patients had at least one IA CS injection into the subtalar joint of the ankle requiring arthrodiastasis.

All patients were treated with MTX, with a mean time from diagnosis to treatment of 3.8 years (range 0.18–7.08 years), and seven of eight patients received biologic treatment. The mean number of biologic agents in those treated was two (range zero to five). The mean time to biologic was 6.44 years (range 4.4–11.9 years).

### Patient- and clinician-reported outcome measures

The mean duration of frame application was 3.3 months (range 2.75–5.5 months), and the mean duration of patient follow-up post-frame removal was 19.8 months (range 12–36 months). No patients were lost to follow-up.

Complications during treatment included three patients with superficial pin-site infections, which were treated successfully with oral antibiotics. There were no re-admissions other than elective admissions for frame removal at the end of the distraction period.

Individual and mean pre- and post-operative differences in scores are shown in Tables 2 and 3, respectively. At all times measured post-frame removal, pain scores were improved from their pre-operative levels. Immediately post-frame removal, the mean pain score improved by 54% (*P *<* *0.05), from 8.2 to 3.8 (range, 1–6). This improved marginally at 6 months post-frame removal by 56% to 3.6 (range, 0–7) and was significantly different from pre-operative levels (*P *<* *0.05). Twelve months post-frame removal, pain scores deteriorated to a mean of 5.8 (range, 0–10) (*P *<* *0.05), but there was still a 29% (*P *<* *0.05) improvement from pre-operative levels. Patients reported that the mean duration of pain improvement after frame removal was 11 months (mean 14.3 months post-index procedure). This varied markedly between patients, with a range of 3–22 months.

**Table 2:** Individual patient pain, functional and clinical outcome scores pre- and post-surgery

	Visual analog scale of pain				OxAFQ-C		OxAFQ-P		AOFAS ankle–hindfoot scale	
Case	Pre-operative	3 months post-operative	6 months post-operative	12 months post-operative	Pre-operative	12 months post-operatie	Pre-operative	12 months post-operative	Pre-operative	12 months post-operative
1	10	4	7	9	4	42	15	30	15	68
2	10	1	0	10	1	19	12	16	17	85
3	10	2	4	9	1	17	12	16	7	73
4	8	6	4	4	5	44	24	44	24	69
5	7	6	2	3	4	42	21	43	38	84
6	10	6	4	5	0	25	4	6	15	81
7	4	6	0	0	6	47	21	49	62	95
8	7	2	4	4	4	44	29	39	47	77
9	8	1	7	8	0	6	4	36	30	57

AOFAS: American Academy Orthopaedic Foot and Ankle Society;OxAFQ-C: Oxford Ankle Foot Questionnaire-Child; OxAFQ-P: Oxford Ankle Foot Questionnaire-Parent.

**Table 3:** Patient pain, functional scoring by Oxford Ankle Foot Questionnaire-Child, Oxford Ankle Foot Questionnaire-Parent and American Academy Orthopaedic Foot and Ankle Society ankle–hindfoot scale

Test	Mean pre-operative (range)	Mean post-operative (range)	Difference (%)	*P*-value
3-month VASP	8.2 (4–10)	3.8 (1–6)	−4.4 (54)	<0.05
6-month VASP	8.2 (4–10)	3.6 (0–7)	−4.6 (56)	<0.05
12-month VASP	8.2 (4–10)	5.8 (0–10)	−2.4 (29)	<0.05
12-month OxAFQ-C	19.9 (4–32)	32.2 (6–47)	+12.3 (62)	<0.05
12-month OxAFQ-P	15.8 (4–29)	28.4 (6–44)	+12.6 (80)	<0.05
12-month AOFAS ankle–hindfoot scale	28.3 (7–62)	76.6 (57–95)	+48.3 (171)	<0.05

AOFAS: American Academy Orthopaedic Foot and Ankle Society;OxAFQ-C: Oxford Ankle Foot Questionnaire-Child; OxAFQ-P: Oxford Ankle Foot Questionnaire-Parent; VASP: visual analog scale of pain.

The mean AOFAS ankle–hindfoot scale pre-operative score was 28.3 (range, 7–62). This improved by a mean of 48.3 (171%) to 76.6 (range, 57–95) at 12 months post-frame removal (*P *<* *0.05). All patients improved their AOFAS ankle–hindfoot scale score at this stage post-operative.

**Fig. 3.**
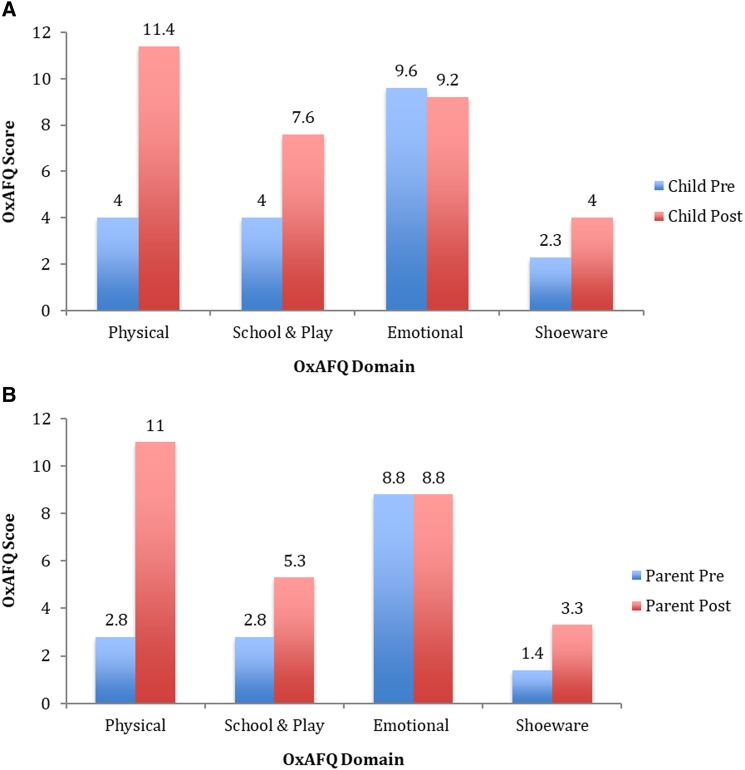
Domain scores shown pre-surgery and at 12 months post-frame removal (**A**) Oxford Ankle Foot Questionnaire-Child (OxAFQ-C). (**B**) Oxford Ankle Foot Questionnaire-Parent (OxAFQ-P).

Both OxAFQ-C and OxAFQ-P demonstrated comparable pre-operative mean scores of 19.9 (range, 4–32) and 15.8 (range, 4–29), respectively. This was mirrored by the 12 month post-frame removal scores of 32.2 (range, 6–47) and 28.4 (range, 6–44) in child and parent, respectively. The parent score demonstrated a greater improvement by a mean of 12.6 (80%) compared with the child score of 12.3 (62%); however, both improvements were statistically significant (*P *<* *0.05). Analysis of the average OxAFQ scores when broken down into the physical, school & play, emotional and shoeware domains can be seen in Fig. 3. The largest mean difference of 7.4 and 8.2 in child and parent scores, respectively, was shown in the physical domain. This was followed by the school & play and shoeware domains, while the emotional score deteriorated marginally in the child score (0.4) but showed no change in the parent score.

Children and parents were questioned regarding whether they were satisfied with arthrodiastasis as a treatment given the significant physical and emotional investment they had made, and whether they would be prepared to go through it again. After six of nine (67%) surgeries, six (67%) children (six ankles) said they were satisfied and that they would undergo the procedure again for the benefit achieved, and eight of nine (89%) parents (eight ankles) said they were satisfied and that they would have their child undergo arthrodiastasis again.

At the time of writing, two patients (three ankles) have undergone further surgical procedures, including arthrodesis (one ankle) and arthroplasty (two ankles). Both reported satisfaction with the procedure and for the period of benefit it gave them. During the period of follow-up, patients had an overall satisfaction with treatment outcome for an average period of 13.3 months (range, 4–20 months) post-frame removal. Beyond this, satisfaction deteriorated as a reflection of worsening pain and function.

Subsequent to this cohort reported, we have treated an additional six patients with arthrodiastasis of the ankle, and of these, one patient has subsequently undergone arthroplasty and one patient arthrodesis.

## Discussion

### Surgical arthrodiastasis of the ankle in JIA

Joint destruction is not a new finding in JIA. Despite recent advances in treatment options for JIA, destructive arthropathy remains a significant challenge. We have clear evidence from discussions within our networks that it is not a totally eradicated problem, although the incidence is likely to be reduced in current generations of patients. We report outcomes in this cohort of eight patients and note that we have subsequently treated a further six patients with surgical arthrodiastasis of the ankle to emphasize that we do not believe this complication of JIA is eradicated, and this report is therefore of relevance to paediatric rheumatologists today.

A similar pattern of ankle disease to the one in this cohort has been described previously [15]. We report the first series of patients with JIA and ankle damage treated by surgical arthrodiastasis by means of an Ilizarov frame and the first non-joint-sacrificing approach to this situation. All patients had radiological evidence of increased joint space, and improvement in pain and function post-operatively, but this was not maintained in all patients. Such improved outcomes post-operatively are the result of restoration of physiological positioning of the ankle joint. Without regeneration of cartilage, however, arthrodiastasis will provide only a temporary mechanical benefit that will delay but almost certainly not eradicate the need for additional surgery. Arthrodiastasis is joint-preserving surgery that might provide a window of opportunity for stem cell therapies to regenerate cartilage surfaces if the efficacy of such technology could be established in future clinical trials. Arthroplasty and arthrodesis are ultimately joint-sacrificing procedures.

Surgical treatment of ankles damaged by JIA is a complex dilemma. Given that surgical treatments have not been described in JIA, there is only evidence from other clinical scenarios, such as adults with OA [12] or joint damage owing to trauma in children [16].

In our cohort, arthrodiastasis provided at least short-term significant patient-reported benefit measured objectively by the AOFAS ankle–hindfoot scale score and subjectively by the visual analog scale of pain and the OxAFQ for parent and child. Our series also demonstrates proof of concept that arthrodiastasis of the damaged ankle joint in JIA re-created joint space in the short to medium term and reduced pain. We also found that the younger the patient treated with ankle distraction, the better the outcome and the longer the duration of benefit they received.

Ankle joint arthrodiastasis is by no means a simple option. Not only does it require significant physical and psychological commitment from the patient, but also the expertise of a multidisciplinary team with experience in applying and managing post-operatively the ring external fixator systems. Given that the emotional burden of long-term JIA in patients with destructive disease is likely to be high, this might also have had a negative impact on some post-operative outcome scores, but we are unable to quantify this.

All patients undergo a formal pre-operative assessment by a team of specialist nurses and physiotherapists and, where necessary, a psychologist. This serves to minimize the impact of the intervention and ensures that the necessary multidisciplinary team is in place before surgery. Patients and their family are thoroughly counselled about the procedure and the likely impact. The patients typically have exhausted all other options and are therefore willing to accept the challenges associated with the procedure. The two patients who reported they would not have the procedure again had very advanced disease and had symptom improvement for the shortest periods. On balance, they felt that the duration of temporary symptom improvement was not worth the physical and emotional cost of the procedure.

### Natural history of JIA and aetiopathogenesis of destructive arthropathy

Persistent inflammation of the synovium causes damage, with loss of the joint space, subluxation and bony osteophyte formation, leading to sclerosis and erosive disease. In the ankle, there flattening of the talar dome, followed by subluxation and eventual joint collapse. Joint collapse is characterized by anterior displacement of the talus subsequent to destruction of the tibial epiphysis. It is anticipated that access to newer medical technologies, such as biologic drugs to control inflammation, will reduce, but might not eradicate, this devastating complication of JIA.

### Anatomical/mechanical factors

It is of note that the ankle is particularly susceptible to erosive damage in JIA. Load-bearing is a predisposing factor. We speculate with regard to the posterior slip of the tibia on the talus that stiffness of the ankle joint causes an increase in sub-talar/mid-foot pronation and this, in combination with the lack of dorsiflexion in the stance phase of gait, causes an internal/posterior movement of the tibia in relationship to the talar dome.

Improvement in disease activity induced by medical treatment might maintain levels of physical function, but if there is sub-clinical or low-grade disease, weight-bearing and postural changes at the ankle may ultimately accelerate damage. This highlights the need for clinical vigilance and a coordinated orthopaedic/rheumatology approach, with a focus on assessments such as gait analysis to understand load-bearing and the use of appliances to shift load off the ankle complex if the joint becomes deranged.

### Disease classification

Ankle destruction has manifested in a range of JIA subtypes, including those classified with persistent oligoarthritis (two of eight in this cohort). It is recognized that children with oligoarthritis are unlikely to follow a benign disease course if the wrist or ankle is involved or if inflammatory markers are elevated [17]. The more benign and less destructive course seen in children with oligoarthritis manifested by single knee involvement in JIA is likely to have influenced treatment approaches historically such that, for patients presenting with oligoarticular ankle disease, there might have been anxiety regarding treatment with DMARDs/biologics early in disease course. We recognize that the disease course in pre-school-age girls with oligoarthritis affecting the knee is significantly different from that of children with oligoarthritis affecting the ankle or wrist. A revised biologic classification of JIA is needed to define the diseases better, predict outcomes and stratify treatment strategies [18].

### Medical treatment factors

Persistent disease activity ultimately leads to destructive disease. We acknowledge that it is relevant that four of eight patients had a delay to treatment with MTX of >6 years, and a mean delay to starting biologic treatment of 6.4 years. This would now be considered historical practice. Such delay was not, however, seen in all patients within this cohort; with two of eight patients were treated with MTX within 3 months of diagnosis, implying that other factors might be involved.

### IA CS injections

IA CS injection with TH is standard of care for induction of remission and/or rescue therapy in JIA. IA CS injection can induce local complications (s.c. atrophy, periarticular calcification, crystal-induced synovitis, avascular necrosis of bone, Cushingoid syndrome, septic arthritis and anaphylaxis [19–22]). All patients in our cohort had evidence of extra-articular soft tissue calcification, a pathological tissue reaction of some concern that we do not believe is reflected adequately in the literature. This is demonstrated on plain radiographs despite the predominant use of image intensifier and contrast injection to ensure correct needle placement. We therefore do not believe that incorrect placement of the needle has predisposed this cohort to less effective response to IA CS injection and hence greater risk of destructive arthropathy owing to inadequate efficacy of local IA CS injection. We note that historical reports have documented much lower rates of peri-articular calcification, but such outcomes have not been reported in a systematic or standardized manner over a prolonged period, such as in our cohort [20]. There remain no definitive data to recommend a change in practice from the use of TH for IA CS injection beyond introduction of systemic immunomodulatory drugs early in the disease course if susceptible joints, such as the ankle and subtalar joint, are involved, but we believe the finding of soft tissue calcification warrants further research into potential mechanisms of tissue injury after IA CS injection.

### Limitations of this study

We acknowledge that there are challenges to the interpretation of single-centre descriptive cohort studies using small numbers of patients, and we cannot validate against outcomes from different centres in the same clinical context. In support of our methodology, we used validated patient- and clinician-reported outcome measures and do not believe that we have over-estimated the effect of the surgical intervention. We believe that our patient selection criteria for surgery and methodology of recording outcomes are robust, and it would be desirable to apply the same criteria to future multicentre studies. Such collaboration will be facilitated by the use of a novel core dataset (‘Capture-JIA’) in the UK, which is to be collected across multiple providers of paediatric rheumatology care [23].

### Conclusion

The occurrence of ankle destruction in JIA has been described historically, but there is a sense within our group and the wider UK paediatric orthopaedic and rheumatology community that this serious complication still occurs. The aetiology is multifactorial and is driven predominantly by disease activity and compounded by load-bearing. All clinicians treating JIA should be vigilant for inflammation of the ankle and subtalar joint, and early aggressive medical treatment is essential.

Arthrodiastasis by means of an Ilizarov frame has been shown to be safe and well tolerated in this cohort, and in the short term to increase joint space and improve patient/parent disease outcome measures, including pain. This technique might provide a window for novel regenerative treatments and allow for irreversible surgical procedures to be delayed until a more suitable age or skeletal maturity.

*Author* *contributions*: G.C.: study design, data collection and analysis, manuscript preparation; C.P.: study design, manuscript preparation; L.M.: study design, manuscript preparation; K.M.: manuscript preparation; S.B.-F.: data collection, analysis, manuscript preparation; S.R.: data collection, analysis, manuscript preparation; R.W.: data collection, analysis, manuscript preparation; A.H.: data collection, analysis, manuscript preparation; C.L.: data collection, analysis, manuscript preparation; N.B.: data collection, analysis, manuscript preparation; I.R.: manuscript preparation; L.J.: study design, manuscript preparation.

*Funding*: No specific funding was received from any funding bodies in the public, commercial or not-for-profit sectors to carry out the work described in this manuscript.

*Disclosure statement*: The authors have declared no conflicts of interest.
